# Supplementary material for: Polyketide synthase gene clusters in the ericoid mycorrhizal fungus Oidiodendron maius: genome-wide prediction, expression profiling, and functional characterization of knockout mutants
Source: Front Plant Sci. 2026 Sep 16;17:1924430. doi: 10.3389/fpls.2026.1924430 (PMC13623903; doi:10.3389/fpls.2026.1924430)
Supplement: Supplementary file 1 [file SupplementaryFile1.zip › Supplementary Materials/Supplementary Folder 1.zip!/Oidma1_AssemblyScaffolds_Repeatmasked/knownclusterblast/cluster38/jgi.p_Oidma1_148477_mibig_hits.html]

| MIBiG Protein | Description | MIBiG Cluster | MiBiG Product | % ID | % Coverage | BLAST Score | E-value |
| --- | --- | --- | --- | --- | --- | --- | --- |
| AFT91387.1 | EcdD | BGC0000338 | Echinocandin\_B\_biosynthetic\_gene\_cluster | 29 | 105.681818182 | 199 | 8.7e-51 |
| AAQ21378.1 | QbsN | BGC0000925 | Quinolobactin\_biosynthetic\_gene\_cluster | 26 | 61.9318181818 | 50 | 5e-06 |
